# Glucocorticoid-Mediated Extracellular Matrix Regulation: Implications for Precision Therapy

**DOI:** 10.3390/biomedicines13061282

**Published:** 2025-05-23

**Authors:** Yinghua Zhu, Yuping Zhang, Ju Shao, Ligang Jie

**Affiliations:** Department of Rheumatology and Clinical Immunology, Zhujiang Hospital, Southern Medical University, Guangzhou 510515, China; zhuyinghua_smu@163.com (Y.Z.); eileenaa@smu.edu.cn (Y.Z.); shaoju1260975454@163.com (J.S.)

**Keywords:** glucocorticoids (GCs), extracellular matrix (ECM), rheumatic diseases, fibroblasts, precise treatment

## Abstract

Glucocorticoids (GCs) have revolutionized the treatment of multidisciplinary diseases. Recently, its role in severe infectious diseases has been revisited and discussed since the COVID-19 pandemic. Previous research and discussions have focused more on their anti-inflammatory effects and impact on the immune system, with limited study on other aspects of their action and mechanisms. In recent years, it has been discovered that glucocorticoids can regulate the extracellular matrix by influencing the cellular microenvironment and processes such as fibrosis, thereby exerting regulatory effects on diseases. This article summarizes current research on GC-mediated extracellular matrix (ECM) remodeling. It emphasizes the dual role of the ECM as a therapeutic target and a source of biomarkers, and identifies molecular mechanisms and potential biomarkers for precise glucocorticoid therapy, such as type I collagen (PRO-C1), type III collagen (PRO-C3), fibrillin-C (FBN-C), and type III collagen degradation (C3M). These findings may also contribute to the development of more precise new drugs.

## 1. Introduction

The therapeutic application of glucocorticoids (GCs) in clinical medicine originated in the late 1950s, representing a revolutionary advancement in treating inflammatory and autoimmune disorders. Subsequent decades have yielded extensive research into their pharmacological effects and molecular mechanisms [1,2,3]. GCs have been widely utilized in COVID-19 treatment due to their anti-inflammatory and immunosuppressive properties, with multiple studies supporting their efficacy in critically ill patients. For instance, a systematic review and network meta-analysis demonstrated that GCs significantly reduce mortality, decrease the need for mechanical ventilation, and improve clinical outcomes in COVID-19 patients [4]. Single-cell RNA sequencing analyses further revealed that GCs suppress type I and II interferon response pathways, while IL-6-associated signatures are additionally downregulated by tocilizumab [5]. A retrospective cohort study also reported that GCs markedly enhance 30-day recovery rates in severe COVID-19 cases [5]. However, their use in non-hypoxemic patients may elevate mortality risks, as evidenced by a meta-analysis showing significantly higher mortality rates in GC-treated individuals compared to controls within this subgroup [6]. While the therapeutic application of GCs in severe and critical COVID-19 has gained broad acceptance [7,8,9,10], their administration during early or mild disease stages remains contentious, necessitating further research to establish optimal treatment protocols and dosing regimens [6,8]. Additionally, individual variability in treatment response and the potential for adverse effects must be carefully considered in clinical decision making [11].

Despite extensive use of GCs and their well-established global anti-inflammatory effects, detailed mechanistic insights—particularly regarding their cell type-specific and context-dependent actions—remain insufficiently explored. In addition to their anti-inflammatory and immunosuppressive properties, GCs critically influence extracellular matrix (ECM) remodeling and cellular microenvironment dynamics.

The ECM is a complex and dynamic component that not only provides structural support to cells but also plays a crucial role in cellular communication, differentiation, and migration [12,13]. In the realm of precision medicine, the ECM is recognized for its critical role in the pathogenesis and progression of various diseases [14]. It serves as a critical determinant in wound healing [15], fibrosis [16], arthritis [17], and cancer metastasis [18]. Understanding the intricacies of ECM dynamics creates opportunities for targeted therapies, enabling more personalized and effective treatment strategies [19].

GCs–ECM interactions constitute a pivotal therapeutic mechanism. GCs modulate ECM remodeling through fibroblast activity, collagen synthesis, and matrix metalloproteinases (MMPs) regulation [20,21]. These bidirectional regulatory effects position the ECM as a potential biomarker for balancing GCs efficacy and adverse effects in precision therapies.

**Figure 1:**
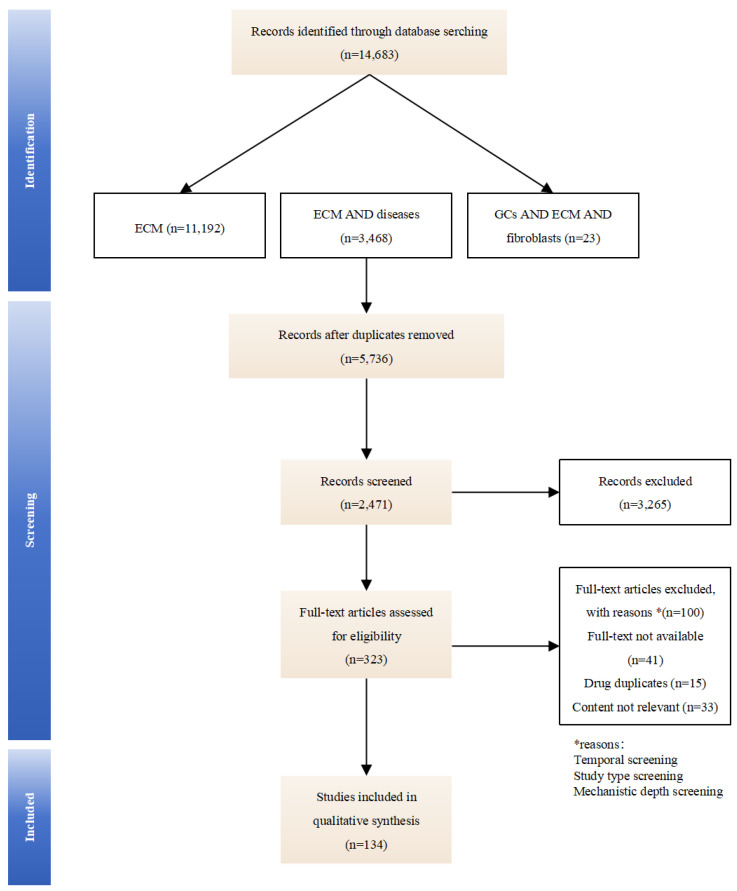
An overview of the studies collection for this study.

This review synthesizes literature from PubMed, Web of Science, and Google Scholar using the following paired search terms: “extracellular matrix”, “extracellular matrix” with “arthritis, fibrosis, cancer, wound healing”, and “glucocorticoids” with “extracellular matrix, fibroblasts”. We evaluate GCs-induced compositional changes in the ECM and their implications for therapeutic optimization and side-effect mitigation, aiming to clarify how GCs dynamically remodel the ECM and identify ECM-related biomarkers for optimizing therapeutic outcomes. By mapping these mechanisms, we seek to provide insights for precision dosing and targeted drug development (an overview of the studies collection for this study is shown in Figure 1).

## 2. What Is the ECM and Its Role in Diseases

The ECM is an intricate network of proteins and polysaccharides that provides structural and biochemical support to the surrounding cells. It is not a static entity; rather, it dynamically interacts with cells, profoundly influencing their behavior and the tissue’s overall function.

### 2.1. Main Structure of the ECM

The ECM is a complex three-dimensional network structure secreted by cells, primarily composed of two functional components: fibrous proteins and a hydrated ground substance. Fibrous proteins, including collagens, elastin, fibronectin, and laminins, form a cross-linked network that confers tensile strength and elasticity to tissues. The hydrated ground substance, consisting of proteoglycans (such as chondroitin sulfate) and glycosaminoglycans like hyaluronic acid, forms a gel-like structure through highly hydrophilic molecules, mediating compressive resistance and regulating tissue osmotic pressure [22,23]. Beyond providing structural support to cells, the ECM integrates bioactive molecules such as growth factors, thereby synergistically regulating cell migration, proliferation, differentiation, and tissue homeostasis through biochemical and biomechanical signals [24].

### 2.2. Variability of the ECM Across Tissues

ECM composition exhibits marked tissue specificity, reflecting unique biomechanical demands [25]. The ECM in cartilage predominantly contains collagen II and aggrecan proteoglycans for shock absorption [26,27], whereas the ECM in skin relies on collagen I and elastin networks for dermal flexibility [28]. Pulmonary ECM predominantly contains heparan sulfate, chondroitin sulfate, and hyaluronic acid, which regulate alveolar elasticity and gas exchange efficiency [29]. Renal function critically depends on specialized basement membrane components within the kidney ECM that govern filtration selectivity [30], while mammary gland ECM orchestrates epithelial cell polarization and lactation through dynamic collagen–proteoglycan networks [31]. The liver ECM, primarily composed of collagen, fibronectin, laminin, and proteoglycans, provides structural support, promotes cell adhesion and growth, and regulates growth factor activity, forming a dynamic network essential for liver function and regeneration [32]. These compositional diversities enable the ECM to structurally reinforce tissues while biochemically fine-tuning cellular responses.

Pathological states drive ECM compositional remodeling. In fibrotic diseases and osteoarthritis, dynamic ECM remodeling manifests as excessive deposition or degradation of matrix components, culminating in loss of structural integrity and function [33,34,35,36,37]. Clinically administered GCs (e.g., dexamethasone)—mainstays in connective tissue disease management—induce ECM alterations that exhibit dual therapeutic and adverse effect signatures, necessitating mechanistic dissection.

### 2.3. Mechanisms of ECM Alteration in Diseases

#### 2.3.1. Arthritis

**Figure 2:**
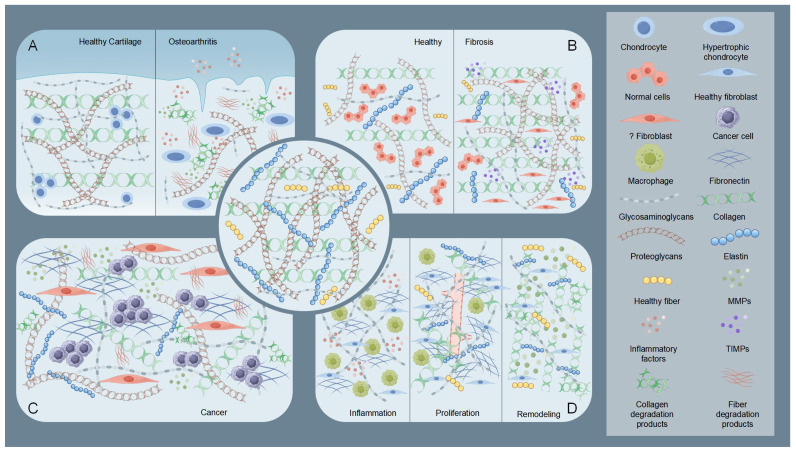
Schematic illustration of the state of the extracellular matrix (ECM) under pathological conditions. The central part is the normal ECM. (**A**) Taking osteoarthritis as an example, the diagram shows a comparison of healthy cartilage ECM with osteoarthritic cartilage ECM. In osteoarthritic cartilage ECM, elevated MMPs and inflammatory factors degrade collagen and proteoglycans. Chondrocytes, influenced by these degradation products and inflammatory factors, undergo hypertrophy and lose their fundamental functions. (**B**) Comparison of ECM in healthy tissue and fibrotic tissue. In fibrotic tissue, abnormal fibroblasts and tissue inhibitor of metalloproteinases (TIMPs) increase, leading to collagen and proteoglycan deposition. (**C**) Diagram showing tumoral ECM, part of the TME. Here, CAFs trigger a marked rise in matrix components, especially adhesive glycoproteins like fibronectin. This action enhances the ability of tumor cells to adhere, migrate, and invade. (**D**) During the three stages of wound healing, the ECM undergoes significant changes. In the inflammatory phase, inflammatory factors and macrophages are the main components. During the proliferative phase, collagen and fibronectin levels increase and begin to arrange in a regular pattern, and new blood vessels form. In the remodeling phase, matrix components are altered by MMPs to reduce scar overgrowth. By Figdraw (https://www.figdraw.com).

Osteoarthritis (OA) exemplifies the critical role of ECM dysregulation in arthritis pathogenesis. Osteoarthritis involves structural degradation of articular cartilage ECM, characterized by disrupted equilibrium between collagen/proteoglycan synthesis and catabolism [38,39] (Figure 2). Elevated MMPs activity coupled with reduced tissue inhibitor of metalloproteinases (TIMPs) expression drives pathological ECM degradation [20], leading to progressive cartilage erosion, joint dysfunction, and pain [17,40]. Inflammatory cytokines (e.g., IL-1β, TNF-α) stimulate chondrocyte-mediated MMPs production, which targets collagen II and aggrecan [40,41,42]. Insufficient compensatory ECM synthesis by chondrocytes accelerates irreversible cartilage loss [17]. This contrasts with rheumatoid arthritis (RA), where MMP-generated ECM neoepitopes perpetuate autoimmune responses [43].

#### 2.3.2. Fibrosis

Fibrosis features pathological ECM overaccumulation, causing tissue scarring and functional impairment (Figure 2). Chronic injury activates myofibroblasts that overproduce collagen I and other ECM components, disrupting organ architecture [44]. The resultant stiffened ECM establishes a self-sustaining fibrotic cascade via mechanotransduction pathways [45,46]. In hepatic and pulmonary fibrosis, this aberrant remodeling progressively compromises organ function through architectural distortion and altered cellular signaling [45,46].

#### 2.3.3. Cancer

The ECM serves dual roles as a physical constraint and tumor-promoting scaffold in cancer progression [47]. Early-stage tumors are restricted by dense ECM barriers, which are later remodeled via tumor-secreted proteases (e.g., MMPs) and cancer-associated fibroblast (CAF)-derived factors [48,49,50] (Figure 2). Modified collagens (e.g., linearized collagen I) and proteoglycan-rich microenvironments facilitate invasion, metastasis, and immune evasion [49,50,51]. CAF-mediated ECM alterations further promote angiogenesis and activate oncogenic signaling pathways (e.g., PI3K/AKT) [50,51,52].

#### 2.3.4. Wound Healing

During the four phases of wound healing (hemostasis, inflammation, proliferation, and remodeling), the ECM exhibits dynamic regulatory characteristics [53] (Figure 2). The ECM not only serves as a structural scaffold but also coordinates cell behavior through its components (such as fibronectin, hyaluronic acid, and collagen) and signal transmission. During the inflammation phase, the deposition of temporary ECM components (e.g., fibronectin and hyaluronic acid) initiates macrophage polarization and inflammation regulation [54,55,56]. In the proliferation phase, enhanced ECM synthesis (e.g., collagen III and fibronectin fibrillation) supports angiogenesis and granulation tissue formation [56,57,58]. During the remodeling phase, collagen remodeling (replacement of type III with type I) mediated by MMPs and regulation by matrix proteins (e.g., decorin) reduce scarring [59,60]. Studies have shown that ECM dynamics are closely related to wound types (e.g., diabetic wounds, burns). And wound healing often requires intervention to accelerate tissue regeneration and prevent complications [61]. For instance, delayed ECM formation in diabetic wounds requires exogenous supplementation with biomaterials (e.g., OHA-CMC hydrogels) [54].

## 3. GC-Mediated Fibroblasts Regulation of ECM Dynamics

### 3.1. Fibroblasts as Central Effectors in ECM Homeostasis

Fibroblasts serve as the principal cellular mediators of ECM synthesis and remodeling during physiological repair and pathological fibrosis [59,62]. Through coordinated biosynthesis of collagens and structural glycoproteins, these cells maintain tissue integrity while dynamically responding to microenvironmental cues [44]. During wound healing, fibroblasts orchestrate granulation tissue formation via ECM deposition and contractile activity, whereas dysregulated activation drives pathological scarring through excessive ECM accumulation.

### 3.2. Inflammation-Induced Fibroblast Phenotypic Switching

In the context of inflammation and fibrosis, fibroblasts undergo phenotypic changes that alter their function [59]. They can transform into myofibroblasts, characterized by increased contractility and ECM production. This transformation is often driven by cytokines and growth factors released during inflammation [45,63]. The persistence of myofibroblasts and their continued ECM production can contribute to the pathological fibrosis observed in various diseases [45].

### 3.3. GCs Orchestrate Fibroblast–ECM Dynamics Across Tissue Contexts

GCs are widely used for their anti-inflammatory effects, but their clinical value is often limited by dose-dependent side effects [64]. A key challenge is balancing efficacy with tissue protection, particularly regarding ECM integrity. GCs influence the ECM both indirectly, by modulating immune responses, and directly, by affecting fibroblasts and epithelial cells. During wound healing, GCs suppress inflammatory-phase ECM degradation by downregulating MMP-2 and MMP-9 activity while exhibiting dose-dependent effects on collagen III synthesis—stimulatory at low concentrations but inhibitory at therapeutic doses [63,65,66,67]. The dose-dependent effects of GCs exhibit significant clinical implications across various diseases. According to existing studies, the dosage thresholds of GCs vary considerably among different diseases and research settings. For instance, in RA, studies have shown that low-dose GCs regimens (<5 mg/day prednisone equivalent) do not significantly increase the risk of cardiovascular events (CVE) compared to high-dose regimens (≥10 mg/day prednisone equivalent) [68,69]. However, the administration of high-dose glucocorticoids has been associated with elevated risks of cardiovascular and all-cause mortality [69]. In anti-neutrophil cytoplasmic antibody-associated vasculitis (AAV), low-dose glucocorticoid therapy (<30 mg/day prednisone equivalent) demonstrates comparable efficacy in inducing remission to high-dose regimens (≥30 mg/day prednisone equivalent) while significantly reducing the incidence of infections [70]. These findings collectively indicate that glucocorticoid dosage selection should be individualized based on specific clinical contexts and patient risk profiles.

**Figure 3:**
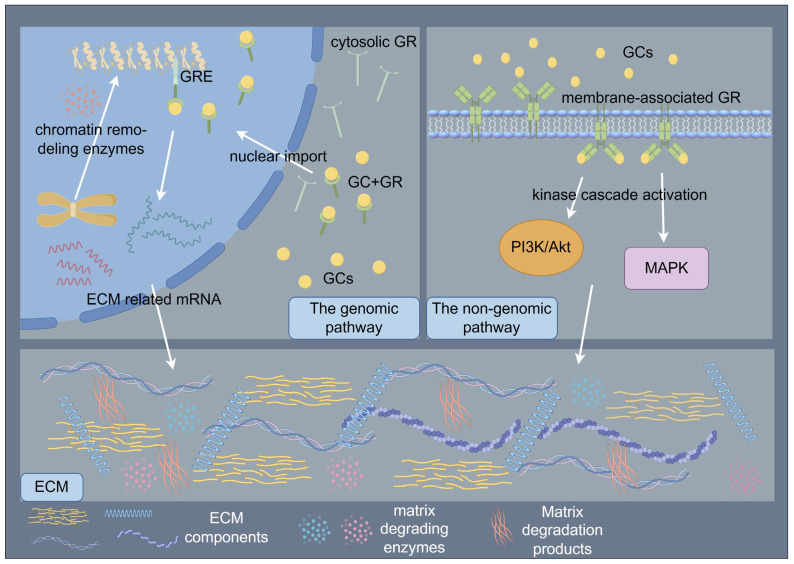
Schematic illustration of glucocorticoids (GCs) signaling orchestrates the regulation of ECM through coordinated genomic and non-genomic mechanisms. The genomic pathway, mediated by cytosolic GR activation, involves ligand-dependent nuclear translocation and subsequent binding to GCs response elements (GREs) in promoter regions, thereby regulating transcriptional programs governing ECM biosynthesis and degradation. The non-genomic mechanisms manifest through rapid membrane-associated GR signaling, which initiates within seconds to minutes via kinase cascade activation, modulating ECM remodeling. By Figdraw (https://www.figdraw.com).

The paradoxical pro- and anti-fibrotic effects of GCs may stem from dose-dependent biphasic regulation of TGF-β signaling and mechanosensitive GC receptor (GR) isoform switching. GC signaling orchestrates ECM regulation through coordinated genomic and non-genomic mechanisms that synergistically modulate cellular behavior and tissue homeostasis [47,71]. The genomic pathway, mediated by cytosolic GR activation, involves ligand-dependent nuclear translocation and subsequent binding to GCs response elements (GREs) in promoter regions, thereby regulating transcriptional programs governing ECM biosynthesis and degradation [71]. Following ligand binding, GRs undergo conformational changes that facilitate their nuclear import, where chromatin remodeling enzymes are recruited to either activate or repress target genes encoding ECM components and modifying enzymes [71]. In contrast, non-genomic mechanisms manifest through rapid membrane-associated GR signaling, which initiates within seconds to minutes via kinase cascade activation (e.g., PI3K/AKT, MAPK pathways), modulating cell migration, proliferation, and ECM remodeling through post-translational regulation of cytoskeletal proteins and matrix metalloproteinases [71]. These temporally distinct yet interconnected pathways converge to dynamically regulate the ECM macromolecular assembly, ultimately determining tissue-specific biomechanical properties and cellular responses to microenvironmental cues [47,71]. (Figure 3) At low doses, GCs activate GR-α to potentiate TGF-β-driven COL1A1 synthesis, whereas higher doses induce miR-29b-mediated TGF-β3 suppression [72,73]. Remodeling-phase ECM maturation is further compromised through GCs-induced MMPs/TIMPs imbalances that impair collagen I crosslinking [20,74]. These multifaceted actions may arise from GR-mediated modulation of AP-1, NF-κB, and TGF-β signaling pathways [75,76,77].

**Figure 4:**
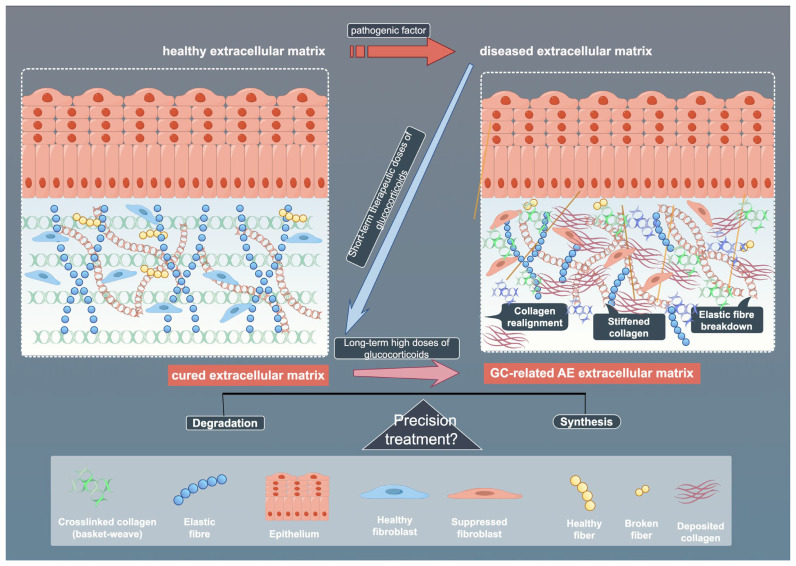
Schematic illustration of ECM across healthy, diseased, and treated states. The figure shows the effects of pathogenic factors and glucocorticoids (GCs) on ECM components, as well as the potential mechanisms of precision therapy. By Figdraw (https://www.figdraw.com).

Fibroblast responses to GCs demonstrate pronounced tissue specificity, shaped by receptor interplay, microenvironmental factors, and metabolic regulation. Tissue-specific differences in 11β-hydroxysteroid dehydrogenase 1 (11β-HSD1) activity modulate local GCs bioavailability and inflammation. Cross-talk between GR and adrenergic receptor (AR) subtypes further diversifies functional outcomes, with GR activation upregulating specific AR subtypes in human fibroblasts [78,79]. Strategies such as selective GR modulators or targeted delivery systems aim to enhance tissue specificity and reduce ECM-related damage, supporting the development of more precise and personalized GCs therapies [80]. Contaminated vocal fold fibroblasts show aberrant dexamethasone sensitivity, emphasizing the need for microbial screening in experiments [81]. Metabolic reprogramming also plays a role, with adipose fibroblasts regulating leptin and cytokine secretion, processes affected by GCs [82]. While beneficial in acute settings, chronic exposure to GCs may disrupt ECM homeostasis and promote fibrosis [83]. Temporal dynamics further refine these effects: acute GCs exposure rapidly activates MMPs (e.g., MMP-2/9 induction in zebrafish within 72 h) alongside compensatory pathways like SGK signaling [84,85], whereas chronic exposure drives irreversible ECM degradation (e.g., perineuronal net disruption) through oxidative stress-inflammatory feedback loops [86]. Certain agents that adversely affect fibroblasts may also disrupt their DNA molecules. For instance, graphene oxide has been shown to exert negative regulatory effects on the cell cycle of embryonic fibroblasts [87]. However, no relevant studies have been observed regarding such DNA-modifying properties in the application of GCs, suggesting that this mechanism may represent a potential therapeutic target in GC-mediated fibroblast regulation. This spatiotemporal modulation of matrix remodeling program responses highlights GCs’ dual therapeutic potential and the need for precision targeting strategies. (Figure 4)

### 3.4. GCs–ECM Interactions in Tumor Microenvironment (TME) Remodeling

GCs critically reshape the TME by modulating ECM dynamics through CAFs activation and immune–ECM cross-talk. GCs enhance CAFs-driven collagen deposition and ECM stiffening via integrin-MMPs signaling, with ovarian cancer models demonstrating DDR2-mediated arginase upregulation that amplifies collagen synthesis [88,89]. This mechanically remodeled ECM promotes tumor invasion while establishing physical barriers to immune infiltration. Thrombospondin-2 (THBS2), a GC-regulated ECM–immune bridge protein, correlates with immunosuppressive TME in gastric/pancreatic cancers through impaired T-cell penetration [90,91].

Clinically, ECM normalization through MMPs inhibition or CAFs depletion enhances chemotherapeutic efficacy in pancreatic cancer, though GCs’ dual roles in immunosuppression and stromal remodeling necessitate tissue-specific strategies [92,93]. Emerging single-cell analyses reveal RUNX2+ myofibroblasts as key GCs–ECM interaction hubs, informing precision approaches to overcome therapy resistance [94,95].

## 4. Precision Treatment of GCs and the ECM as Clues for Its Biomarkers

Precision medicine is a tailored medical approach that integrates an individual’s genetic makeup, environmental factors, and lifestyle characteristics to deliver personalized medical decisions, treatments, and preventive strategies [96]. While there is evidence-based medical support for glucocorticoid therapy, further research is needed to advance precision medicine approaches based on molecular pharmacological mechanisms [77]. For example, a study shows that three-month tapering and discontinuation of long-term, low-dose glucocorticoids in senior patients with RA is feasible and safe, which is based on a placebo-controlled, double-blind tapering after the GLORIA trial [97]. Another real-world research study revealed that GCs are feasibly discontinued with favorable control of disease activity in real-life settings, mostly without short-term flare. However, the withdrawal time is far from reaching the recommended time frame, indicating the gap between real-world practice and current guidelines [98].

### 4.1. Classification and Detection of ECM Biomarkers

ECM biomarkers are broadly categorized based on their biological roles: collagen formation markers, collagen degradation markers, elastin breakdown markers, and MMP/TIMP enzyme profiles [99,100]. Collagen formation markers, including PRO-C1 (type I collagen), PRO-C3 (type III collagen), and PRO-C6 (type VI collagen), reflect ECM synthesis activity [99,100]. In contrast, collagen degradation markers such as C1M (type I collagen degradation), C3M (type III collagen degradation), and TUM (type IV collagen degradation) indicate ECM degradation processes [99,100,101]. Elastin breakdown markers like EL-NE reflect ECM degradation mediated by neutrophil elastase [99]. Additionally, MMPs and TIMPs, such as MMP-2, MMP-9, and TIMP-1, reveal the enzymatic mechanisms of ECM remodeling [43,102,103]. These biomarkers capture dynamic ECM turnover processes, with specific clinical relevance in GCs therapy optimization. For instance, elevated BGN (Biglycan) levels may correlate strongly with GCs-induced osteonecrosis risk, providing a quantitative basis for early intervention [104,105]. Collagen degradation markers such as C3M and TUM further serve as sentinels of ECM destabilization in fibrotic diseases, while elastin degradation products like EL-NE reflect neutrophil-driven tissue damage in chronic inflammatory conditions [99,101].

Advanced detection methods like ELISA and mass spectrometry enable precise quantification, while multi-marker panels (e.g., C6M + Pro-C6 + EL-NE) improve diagnostic accuracy in complex diseases such as COPD [99,100,101]. Emerging platforms further expand clinical utility: the BrdU incorporation lymphocyte steroid sensitivity assay (BLISS) achieves 83% sensitivity in identifying GC-resistant patients by measuring lymphocyte proliferation responses ex vivo, offering a non-radioactive alternative to traditional assays [106]. Three-dimensional organoid platforms now faithfully reconstruct tissue-specific ECM architectures, particularly in ovarian cancer models [107,108], where preserved patient-specific ECM components enable systematic screening of GCs sensitivity and chemoresistance prediction [108].

The bidirectional regulation of ECM components by GCs underscores their biomarker potential. Therapeutic GC doses suppress MMP-9 activity in inflamed joints, preserving collagen integrity and reducing cartilage degradation, whereas prolonged high-dose GC exposure disrupts ECM homeostasis, accelerating skin atrophy through collagen I/III imbalance and MMP-mediated elastolysis [109,110]. This duality positions MMP-9/TIMP-1 ratios as dynamic biomarkers to balance GC efficacy and toxicity. Furthermore, proteomic profiling reveals GCs-induced suppression of fibronectin and fibulin-1 in dermal ECM, while paradoxically upregulating collagen IV via YAP pathway activation in endothelial cells [111,112,113,114,115]. It can be a mechanism exploitable for tissue-specific biomarker development.

### 4.2. ECM-Guided GCs Treatment Strategies

In rheumatic diseases, ECM markers have shown potential for guiding treatment decisions. In RA, transcriptomic profiling of synovial tissue (RNA sequencing and single-cell RNA sequencing), integrated with spatial multi-omics platforms (e.g., 10× Visium), has unveiled fibroblast–T cell interaction networks and dynamic alterations in ECM components, which predict therapeutic responses to GCs [116]. By applying the MITHrIL algorithm to quantify miRNA-regulated immune pathways, combined with gene interaction network analysis (e.g., SOCS2/STAT2 axis), this study identified ECM remodeling-associated biomarkers (e.g., upregulated MMP-3 and downregulated TIMP-1), providing a mechanistic foundation for precision-targeted therapies in RA [117,118]. A study on psoriatic arthritis (PsA) patients found that baseline ECM marker levels can predict the treatment response to guselkumab, an IL-23 inhibitor. Similar strategies may apply to GCs treatment, using ECM markers to identify patient subgroups likely to benefit [43,103,119]. In kidney diseases, human precision-cut kidney slices (PCKSs) models have confirmed that ECM markers like PRO-C1, PRO-C3, and FBN-C reflect fibrosis extent and drug responses [101]. These models can test GCs’ effects on renal ECM remodeling and identify patients likely to benefit from GCs. For pulmonary fibrosis, PRO-C3 and C3M dynamics correlate with disease progression, supporting their use in monitoring GCs effects [100].

Long-term GCs therapy is associated with significant complications requiring vigilant monitoring. Osteonecrosis occurs in 9–40% of adults receiving chronic GCs treatment, independent of osteoporosis development. Adrenal suppression—characterized by hypothalamic–pituitary–adrenal (HPA) axis dysfunction—arises from exogenous GCs exposure, with even short-term high-dose regimens (≥5 days) inducing cortisol insufficiency. Systemic absorption of inhaled/topical GCs and long-acting formulations (e.g., dexamethasone) amplifies this risk, though morning administration may mitigate suppression severity through circadian rhythm alignment [59]. Notably, sex-specific variations in MMPs activity necessitate gender-adjusted biomarker interpretation to optimize GCs dosing [103]. Current research indicates that given the potential adverse effects of GCs, such as skin atrophy and angiogenesis suppression, the exploration of alternative therapeutic strategies has become a critical focus. Selective GR agonists, exemplified by 5α-tetrahydrocorticosterone (5αTHB), demonstrate potential benefits in ECM regulation. Compared to conventional GCs like hydrocortisone, 5αTHB exhibits weaker inhibitory effects on angiogenesis while preserving the expression of genes associated with ECM integrity and inflammatory signaling pathways [120]. However, it should be emphasized that existing studies remain confined to animal models, necessitating further human trials to validate its safety profile and therapeutic efficacy. Concurrently, the development of MMPs inhibitors has advanced, with diverse candidates such as gold nanorods, doxycycline, and natural products under investigation, each operating through distinct mechanisms [121]. Nevertheless, limited understanding of the multifaceted roles of MMPs in disease pathogenesis, coupled with inadequate selectivity of inhibitors, has resulted in the failure of numerous MMP-targeted agents in clinical trials [121,122].

Emerging biomarkers offer solutions for complication management. GCs-induced leucine zipper protein (GILZ) is a GCs-responsive regulator, modulates cellular activation, apoptosis, and inflammation via direct inhibition of NF-κB subunits (p65/p52). It attenuates pro-inflammatory cytokine release and macrophage phagocytosis [123]. This dual functionality positions GILZ as both a predictive biomarker for sepsis outcomes and a therapeutic monitoring target for glucocorticoid regimen optimization.

Understanding how glucocorticoids interact with the ECM at the molecular and cellular levels could lead to more targeted and effective treatments for rheumatic diseases. Healthcare professionals should be aware of these potential biomarkers and management strategies to optimize glucocorticoid treatment plans and minimize adverse patient reactions.

### 4.3. Challenges and Future Directions

Despite the potential of ECM biomarkers in optimizing GCs therapy, key challenges persist. Longitudinal studies are scarce, with most research relying on cross-sectional designs that fail to capture dynamic ECM changes during long-term GCs treatment [99,100,124]. Standardization issues plague biomarker detection, as inconsistent assay protocols and cutoff values hinder clinical translation [99,101,103]. Mechanistic understanding remains incomplete, particularly regarding how GCs selectively modulate specific ECM components like fibronectin versus collagen [43,100,103]. Additionally, the lack of multi-omics integration (e.g., combining ECM profiles with genomic data) limits predictive model accuracy [124,125].

To address these gaps, researchers should prioritize multi-modal models that integrate ECM biomarkers, clinical parameters, and imaging data to predict GCs responses [125,126]. Patient-derived organoids and 3D culture systems offer physiologically relevant platforms for testing GCs–ECM interactions at the individual level [107,108,127]. Emerging technologies such as nanobiosensors and liquid biopsies could enable real-time ECM monitoring with high sensitivity [128,129]. Clinical trials must validate ECM-guided GCs dosing strategies, particularly for diseases with sex-specific ECM remodeling patterns [100,119,129]. Investigating ECM–immune cell cross-talk may reveal novel therapeutic targets to enhance GCs efficacy [130,131,132].

Artificial intelligence (AI) holds transformative potential, with deep learning algorithms analyzing spatial ECM heterogeneity in tissue samples to improve prognostic accuracy [126,133]. These tools could optimize GCs treatment decisions by quantifying structural ECM changes during therapy [126,133].

## 5. Conclusions

The field of precision treatment with GCs in rheumatic diseases is rapidly evolving. Current strategies focus on personalized tapering and adverse effect monitoring, while future developments are likely to involve advanced diagnostics and novel therapeutic agents, representing a shift towards more effective, safer, and personalized GCs therapy. The ECM is a complex network of proteins and other molecules that provides structural and biochemical support to surrounding cells. The ECM can play a role in the precision treatment with GCs, particularly in rheumatic diseases. It can emerge as a critical biomarker reservoir for optimizing GCs therapy, bridging molecular mechanisms with clinical outcomes.

In summary, the ECM is an important factor in the pathophysiology of rheumatic diseases and can influence the effectiveness of GCs therapy. Further research into the ECM’s role could enhance the precision of GCs treatment. It can enable more targeted drug delivery and provide new biomarkers for monitoring disease progression and treatment response.

## Abbreviations

extracellular matrix	ECM
glucocorticoids	GCs
matrix metalloproteinases	MMPs
tissue inhibitor of metalloproteinases	TIMPs
cancer-associated fibroblasts	CAFs
α-smooth muscle actin	α-SMA
GC receptor	GR
rheumatoid arthritis	RA
11β-hydroxysteroid dehydrogenase 1	11β-HSD1
adrenergic receptor	AR
tumor microenvironment	TME
thrombospondin-2	THBS2
type I collagen	PRO-C1
type III collagen	PRO-C3
type VI collagen	PRO-C6
type I collagen degradation	C1M
type III collagen degradation	C3M
type IV collagen degradation	TUM
psoriatic arthritis	PsA
precision-cut kidney slices	PCKS
artificial intelligence	AI
interleukin-1β	IL-1β
interleukin-6	IL-6
interleukin-23	IL-23
tumor necrosis factor-α	TNF-α
phosphatidylinositol 3-kinase	PI3K
protein kinase B	AKT
mitogen-activated protein kinase	MAPK
transforming growth factor β	TGF-β
collagen type II α 1 chain	COL1A1
activator protein 1	AP-1
nuclear factor κ-B	NF-κB
serum and glucocorticoid induced kinase	SGK
discoidin domain receptor	DDR2
runt-related transcription factor 2+	RUNX2+
fibrillin-C	FBN-C
elastin degradation by neutrophil elastase	EL-NE
chronic obstructive pulmonary disease	COPD
biglycan	BGN
5-bromo-2′-deoxyuridine	BrdU
BrdU incorporation lymphocyte steroid sensitivity assay	BLISS
client-side JavaScript MVC framework	MITHrIL
suppressor of cytokine signaling 2	SOCS2
signal transducer and activator of transcription	STAT2
hypothalamic–pituitary–adrenal	HPA
GCs-induced leucine zipper protein	GILZ
glucocorticoid response elements	GREs
5α-tetrahydrocorticosterone	5αTHB
cardiovascular events	CVE
antibody-associated vasculitis	AAV
